# Genome editing and chromosome engineering in plants

**DOI:** 10.1002/tpg2.20352

**Published:** 2023-06-18

**Authors:** Arjun Ojha, Feng Zhang, Gunvant B. Patil

**Affiliations:** ^1^ Institute of Genomics for Crop Abiotic Stress Tolerance, Department of Plant and Soil Sciences Texas Tech University Lubbock Texas USA; ^2^ Department of Plant and Microbial Biology University of Minnesota Saint Paul Minnesota USA

## EDITING PLANT GENOME IN THE POST‐GENOME SEQUENCING ERA

**FIGURE 1:**
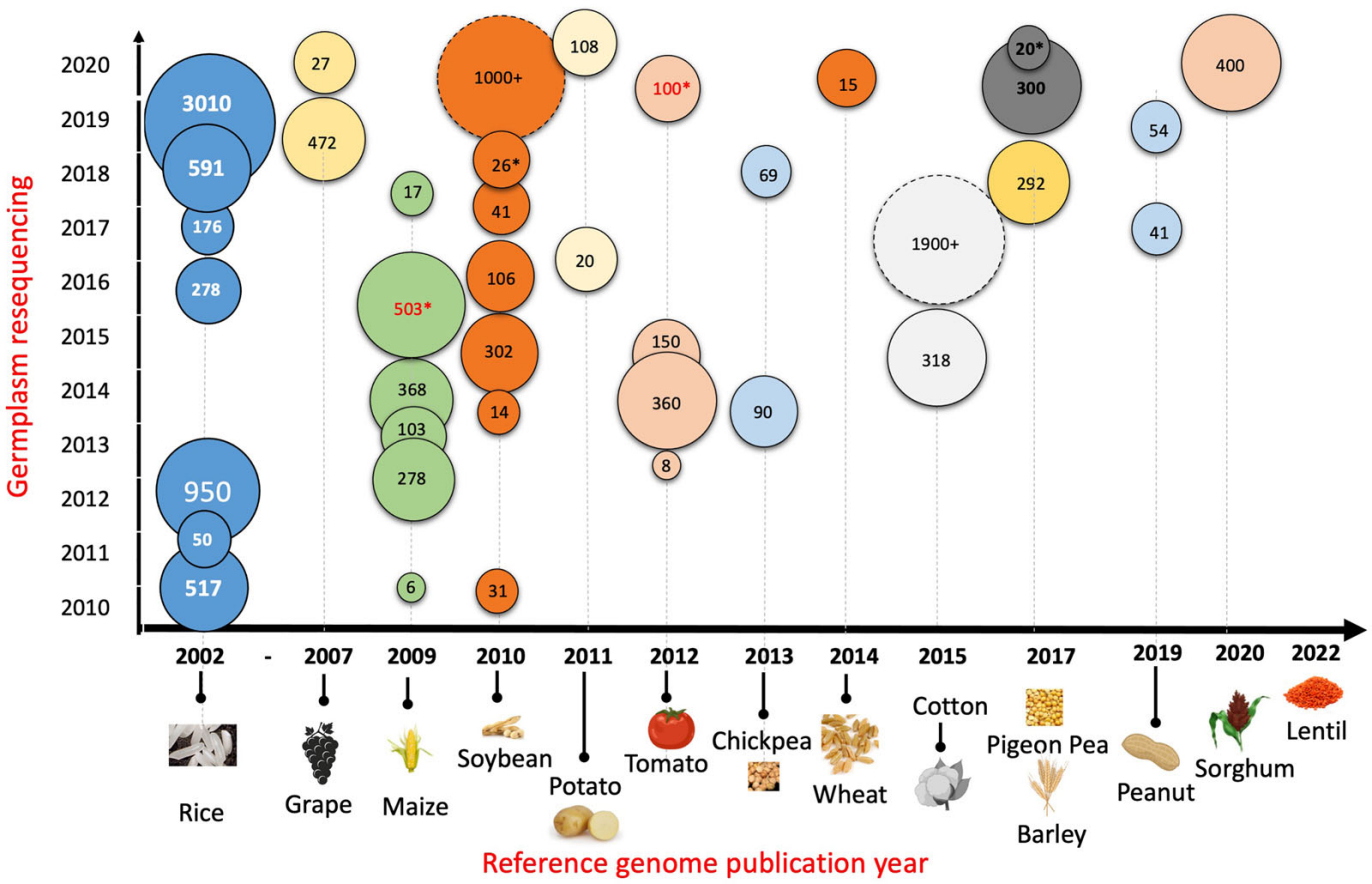
Development of the reference genome and germplasm sequencing resources of some major crops. Circles represent the number of genomes sequence. Asterisk (*) represents pan‐genome sequencing data. Data *source*: Phytozome. Note: Circles are not up to the scale.

In the last two decades, innovations in genomics (genome reading) have advanced crop breeding and have played a central role crop improvement. Since first crop genome (rice) was published, several crop genomes are out in the public domain with an exponential increase in the number of samples per genome, improved genome assembly and development of PAN genomes (Figure 1). The availability of reference, whole genome re‐sequencing (WGRS) and PAN‐genomes are essential to map allelic variants (single base pair to large variations) and have advanced our knowledge toward precise gene discovery, marker development, and trait introgression (Bevan et al., 2017; Kadam et al., 2016; Patil et al., 2016; Valliyodan et al., 2021). WGRS and development of PAN genomes is one of the many payoffs of trait discovery programs and has been conducted in a variety of organism including humans, animals, and several crop species (Figure 1). The dense variation data integrated with other omics platforms (transcriptomics, phenomics, and metabolomics) improves the understanding of phenotype–genotype relationship and essential for marker‐assisted breeding and gene mapping. Several crop‐specific databases have been developed to accelerate the trait mapping, and this information has become an integral part of all aspects of biological research, including basic and applied plant biology (Deshmukh et al., 2021; Matthews et al., 2009). With integrated genomic technologies, researchers can now effectively identify casual genetic variants from wild/landrace relatives. Application of robust and high throughput genome engineering technologies will be essential to understand gene function and achieve targeted modification of desirable agronomic traits in a wide range of plants, especially crop species.

**FIGURE 2:**
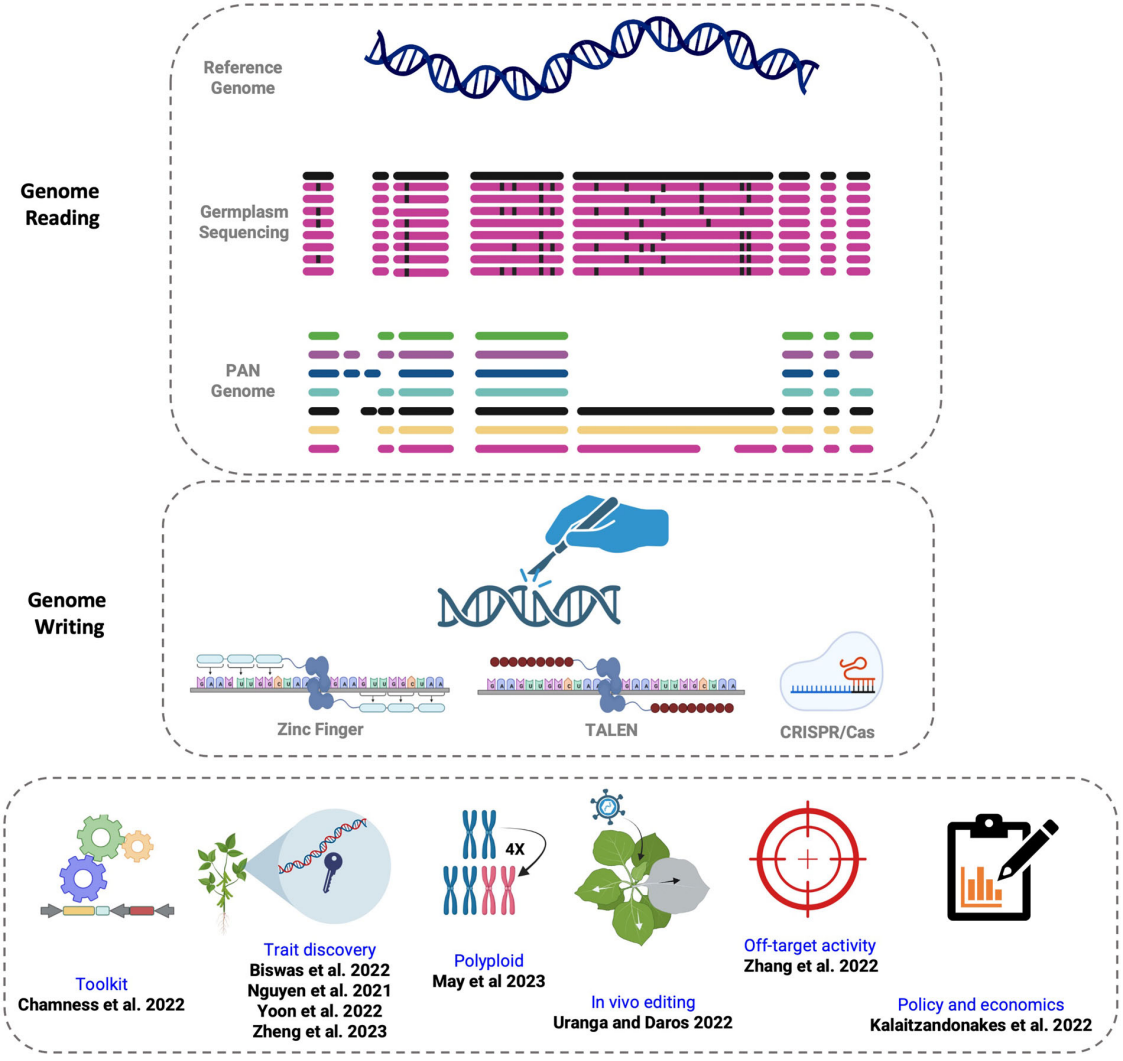
Advances in genome reading and writing in plants.

Over the years, plant genome modification technologies have evolved rapidly from random mutagenesis approaches, such as chemical, physical, or insertional (transposon or T‐DNA mediated) mutagenesis, to more targeted and precise techniques, such as genome editing (Graham et al., 2020). Although random mutagenesis often requires creating large populations for mutant screening without much control on the mutation outcomes, genome editing enables the introduction of a broad range of genetic modifications in a more efficient and controlled manner. In the past three decades, the continuous search for high efficiency and target‐specific genetic engineering technologies lead to the development of several genome editing tools, including meganucleases, zinc‐finger nuclease, TALENs (transcription activator‐like effector nucleases) and more recently CRISPR‐Cas (Vats et al., 2019). The first three genome editing technologies often required laborious efforts to engineer their DNA binding domains for new sequence recognition activities. By contrary, the CRISPR‐Cas technology is directed by the CRISPR guide RNA (gRNA) system to search, bind, and create targeted genetic modifications through RNA–DNA base pairing. Due to its simplicity, robust activity, versatility, and multiplexing capability, the CRISPR‐Cas system has become the reagent of choice for genome editing and has been successfully demonstrated in various plant species to create targeted genetic modifications. Compared to the genome editing systems in animal and mammalian species, plant and crop species present their own opportunities and challenges. In this focus issues, a collection of reviews and research articles were published to tackle several key challenges, including building versatile genome modification toolkits, development of genome editing technologies for polyploid genomes, integration of genome editing with other molecular breeding technologies, trait development through multiplexing genome editing, precision assessment of CRISPR‐Cas genome editing technologies, and current status quo on regulatory landscape of genome editing technologies and products (Figure 2).

Delivery of gene‐editing reagents largely relies of transformation technologies that are time‐consuming and labor intensive. Uranga and Daros (2022) reviewed recent advances in virus induced gene editing (VIGE) and discussed the application of VIGE toolbox with special focus on strategies to achieve tissue culture free delivery of gene‐editing reagents. In addition, gene‐editing regent delivery and coordinated expression in plant cell depend upon streamlined processes to develop efficient vector systems with a wide of collection of building parts, including Cas enzymes, promoters, terminators, and selection/reporter genes. Chamness et al. (2022) reported a universal golden gate‐based cloning toolkit to simplify the assembly of complex constructs for plant genome engineering. These publicly available cloning resources would greatly reduce the bottleneck of technical challenges and accelerate the testing and deployment of new tools. In this special focus issue, using the optimized CRISPR‐Cas9 approach, Biswas et al. (2022) and Nguyen et al. (2021) characterized multiple novel genes involved in resistant starch in rice and N fixation and nodule development in soybean, respectively. In addition, Yoon et al. (2022) applied CRISPR‐Cas9 to develop double haploid technology in switchgrass that could significantly speed up its plant breeding processes. By targeting four functional domains of the CENH3 homolog genes, a number of aneuploid and haploid lines have been developed first time in this species.

In plant genome engineering, however, the polyploid genomes and genetic redundancy from multiple‐member gene families often present additional technical and practical challenges in gene function characterization, trait discovery, and crop improvement. May et al. (2023) reviewed the progress, obstacles, and optimizations that are needed to achieve efficient targeted mutagenesis in polyploids. To address genetic redundancy issues, Zheng et al. (2023) showcased the generation of single, double, and triple knockouts of 11 members in the Cytokinin oxidase/dehydrogenase (CKX) gene family in rice. Although significant functional redundancy was identified among OsCKX gene members in the same phylogenetic clade, their findings revealed the diversified regulating capabilities of OsCKX genes to modulate agronomic traits such as plant height, panicle size, grain size, and grain number per panicle.

In addition to the technical challenges mentioned above, reducing off‐target effect is essential in crop improvement. Zhang et al. (2022) investigated the impact of highly multiplexed CRISPR‐Cas12a system on off‐targeting in rice and identified that simultaneous introduction of multiple double stranded DNA breaks could lead to large chromosome rearrangements. This research is particularly important for the implementation of regulatory policies. To this end, Kalaitzandonakes et al. (2022) first analyzed the economics of genome editing and its potential long‐term effect on crop improvement and agriculture and then discussed the emerging global regulatory policy environment, which would shape the ultimate path for genome editing innovations and overall socioeconomic benefits to the society.

## AUTHOR CONTRIBUTIONS

**Arjun Ojha**: Writing – original draft;Writing – review & editing. **Feng Zhang**: Writing – original draft; Writing review & editing. **Gunvant Patil**: Writing – original draft; Writing review & editing.

## CONFLICT OF INTEREST STATEMENT

The authors declare no conflicts of interest.

## References

[tpg220352-bib-0001] Bevan, M. W. , Uauy, C. , Wulff, B. B. H. , Zhou, J. , Krasileva, K. , & Clark, M. D. (2017). Genomic innovation for crop improvement. Nature, 543(7645), 346–354. 10.1038/nature22011 28300107

[tpg220352-bib-0002] Biswas, S. , Ibarra, O. , Shaphek, M. , Molina‐Risco, M. , Faion‐Molina, M. , Bellinatti‐Della Gracia, M. , Thomson, M. J. , & Septiningsih, E. M. (2022). Increasing the level of resistant starch in ‘Presidio’ rice through multiplex CRISPR‐Cas9 gene editing of starch branching enzyme genes. The Plant Genome, e20225. 10.1002/tpg2.20225 35713092 PMC12806894

[tpg220352-bib-0003] Chamness, J. C. , Kumar, J. , Cruz, A. J. , Rhuby, E. , Holum, M. J. , Cody, J. P. , Tibebu, R. , Gamo, M. E. , Starker, C. G. , Zhang, F. , & Voytas, D. F. (2022). An extensible vector toolkit and parts library for advanced engineering of plant genomes. The Plant Genome, e20312. 10.1002/tpg2.20312 PMC12807070 36896468

[tpg220352-bib-0004] Deshmukh, R. , Rana, N. , Liu, Y. , Zeng, S. , Agarwal, G. , Sonah, H. , Varshney, R. , Joshi, T. , Patil, G. B. , & Nguyen, H. T. (2021). Soybean transporter database: A comprehensive database for identification and exploration of natural variants in soybean transporter genes. Physiologia Plantarum, 171(4), 756–770. 10.1111/ppl.13287 33231322

[tpg220352-bib-0005] Graham, N. , Patil, G. B. , Bubeck, D. M. , Dobert, R. C. , Glenn, K. C. , Gutsche, A. T. , Kumar, S. , Lindbo, J. A. , Maas, L. , May, G. D. , Vega‐Sanchez, M. E. , Stupar, R. M. , & Morrell, P. L. (2020). Plant genome editing and the relevance of off‐target changes. Plant Physiology, 183(4), 1453–1471. 10.1104/pp.19.01194 32457089 PMC7401131

[tpg220352-bib-0006] Kadam, S. , Vuong, T. D. , Qiu, D. , Meinhardt, C. G. , Song, L. i. , Deshmukh, R. , Patil, G. , Wan, J. , Valliyodan, B. , Scaboo, A. M. , Shannon, J. G. , & Nguyen, H. T. (2016). Genomic‐assisted phylogenetic analysis and marker development for next generation soybean cyst nematode resistance breeding. Plant Science, 242, 342–350. 10.1016/j.plantsci.2015.08.015 26566850

[tpg220352-bib-0007] Kalaitzandonakes, N. , Willig, C. , & Zahringer, K. (2022). The economics and policy of genome editing in crop improvement. The Plant Genome, e20248. 10.1002/tpg2.20248 36321718 PMC12807197

[tpg220352-bib-0008] Matthews, D. E. , Lazo, G. R. , & Anderson, O. D. (2009). Plant and crop databases. Plant Genomics, 513, 243–262. 10.1007/978-1-59745-427-8_13 19347656

[tpg220352-bib-0009] May, D. , Paldi, K. , & Altpeter, F. (2023). Targeted mutagenesis with sequence‐specific nucleases for accelerated improvement of polyploid crops: Progress, challenges, and prospects. The Plant Genome, e20298. 10.1002/tpg2.20298 36692095 PMC12806893

[tpg220352-bib-0010] Nguyen, C. X. , Dohnalkova, A. , Hancock, C. N. , Kirk, K. R. , Stacey, G. , & Stacey, M. G. (2021). Critical role for uricase and xanthine dehydrogenase in soybean nitrogen fixation and nodule development. The Plant Genome, e20171. 10.1002/tpg2.20172 PMC12807372 34904377

[tpg220352-bib-0011] Patil, G. , Do, T. , Vuong, T. D. , Valliyodan, B. , Lee, J.‐D. , Chaudhary, J. , Shannon, J. G. , & Nguyen, H. T. (2016). Genomic‐assisted haplotype analysis and the development of high‐throughput SNP markers for salinity tolerance in soybean. Scientific Reports, 6(1), 1–13. 10.1038/srep19199 26781337 PMC4726057

[tpg220352-bib-0012] Uranga, M. , & Daròs, J. A. (2022). Tools and targets: The dual role of plant viruses in CRISPR‐Cas genome editing. The Plant Genome, e20220. 10.1002/tpg2.20220 35698891 PMC12806914

[tpg220352-bib-0013] Valliyodan, B. , Brown, A. V. , Wang, J. , Patil, G. , Liu, Y. , Otyama, P. I. , Nelson, R. T. , Vuong, T. , Song, Q. , Musket, T. A. , Wagner, R. , Marri, P. , Reddy, S. , Sessions, A. , Wu, X. , Grant, D. , Bayer, P. E. , Roorkiwal, M. , Varshney, R. K. , … Nguyen, H. T. (2021). Genetic variation among 481 diverse soybean accessions, inferred from genomic re‐sequencing. Scientific Data, 8(1), 1–9. 10.1038/s41597-021-00834-w 33558550 PMC7870887

[tpg220352-bib-0014] Vats, S. , Kumawat, S. , Kumar, V. , Patil, G. B. , Joshi, T. , Sonah, H. , Sharma, T. R. , & Deshmukh, R. (2019). Genome editing in plants: Exploration of technological advancements and challenges. Cells, 8(11), 1386. 10.3390/cells8111386 31689989 PMC6912757

[tpg220352-bib-0015] Yoon, S. , Bragg, J. , Aucar‐Yamato, S. , Chanbusarakum, L. , Dluge, K. , Cheng, P. , Blumwald, E. , Gu, Y. , & Tobias, C. M. (2022). Haploidy and aneuploidy in switchgrass mediated by misexpression of CENH3. The Plant Genome, e20209. 10.1002/tpg2.20209 35470589 PMC12807150

[tpg220352-bib-0016] Zhang, Y. , Wu, Y. , Li, G. , Qi, A. , Zhang, Y. , Zhang, T. , & Qi, Y. (2022). Genome‐wide investigation of multiplexed CRISPR‐Cas12a‐mediated editing in rice. The Plant Genome, e20266. 10.1002/tpg2.20266 36177842 PMC12806924

[tpg220352-bib-0017] Zheng, X. , Zhang, S. , Liang, Y. , Zhang, R. , Liu, L. , Qin, P. , Zhang, Z. , Wang, Y. , Zhou, J. , Tang, X. , & Zhang, Y. (2023). Loss‐function mutants of OsCKX gene family based on CRISPR‐Cas systems revealed their diversified roles in rice. The Plant Genome, e20283. 10.1002/tpg2.20283 36660867 PMC12807126

